# Salivary Interleukin 1-beta levels and clinical periodontal parameters in habitual naswar users and non-users

**DOI:** 10.12669/pjms.35.3.10

**Published:** 2019

**Authors:** Zaeem Arif Abbasi, Naila Irum Hadi, Adnan Mustafa Zubairi, Mervyn Hosein

**Affiliations:** 1 *Dr. Zaeem Arif Abbasi, BDS., Ziauddin University, 4/B, Sharah-e-Ghalib, Block 6, Clifton, Karachi, Pakistan*; 2 *Prof. Dr. Naila Irum Hadi, Ph.D., M.Phil, MBBS. Islamabad Medical & Dental College, Murree Road, Satra Mile, Islamabad, Pakistan*; 3 *Dr. Adnan Mustafa Zubairi, FCPS (Chem Path), DCP, MBBS., Ziauddin University, 4/B, Sharah-e-Ghalib, Block 6, Clifton, Karachi, Pakistan*; 4 *Prof. Dr. Mervyn Hosein, FFDRCSI, FDSRCSE, FDSRCS, BDS., Ziauddin University, 4/B, Sharah-e-Ghalib, Block 6, Clifton, Karachi, Pakistan*

**Keywords:** Inflammation, Interleukin (IL-1β), Naswar, Periodontitis, Saliva.

## Abstract

**Objectives:**

Aim of our study was to assess the expression of salivary Interleukin 1-beta (IL-1β) and clinical periodontal parameters in *naswar* users and non-users (controls).

**Methods:**

Eighty four individuals (forty-two *naswar* users and forty-two controls) were included in the study which was conducted between August 2017 and May 2018. Salivary IL-1β levels, plaque index (PI), bleeding on probing (BOP), probing depth (PD) and clinical attachment loss (CAL) was assessed in all the participants.

**Results:**

PD of 4mm (p<0.05), PD of 5-6mm (p<0.05), CAL (p<0.001) and levels of salivary IL-1β (p<0.05) were significantly higher among *naswar* users as compared to controls while PI, BOP and number of missing teeth showed no significant difference among the two groups (p>0.05).

**Conclusion:**

Periodontal inflammatory conditions were worse and salivary IL-1β levels were elevated in *naswar* users as compared to controls.

## INTRODUCTION

*Naswar* is categorized as one of the smokeless tobacco products (STPs), the ingredients of which are mainly sundried crushed local tobacco, ash, calcium oxide (slaked lime), and sometimes flavoring agents (e.g., cardamom, menthol) as well as coloring agents (indigo).^1^ It is commonly used in Pakistan, Afghanistan, Iran, Central Asia and South Africa.^1^ *Naswar* usage is gaining popularity as it is now available and being consumed in different parts of the world including England.^2^ It is prepared and wrapped into small plastic bags which even lack safety warnings.^1^ It is used mostly by applying and retaining it in the vestibular cavity adjacent to the buccal or labial mucosa or at times under the tongue.^2^ All the other types of STPs are consumed via chewing but *naswar* is never bitten because of its bad taste.^2^

The tobacco being currently used in *naswar* contains trace amounts of arsenic, beryllium, cadmium, chromium, cobalt, lead, nickel, nitrate, nitrite and tobacco specific nitrosamines (TSNAs).^1^ Along with these substances, addictive nicotine is also found in *naswar*.^3^

The main characteristics of chronic periodontitis are periodontal attachment loss, connective tissue degradation and resorption of alveolar bone. The disease process is initiated by bacterial pathogens however it has been proven that they alone cannot be responsible for the severity of tissue destruction occurring in periodontitis.^4^

Currently accepted theory related to pathogenesis of periodontitis suggests that alteration in host defenses by bacterial and host products stimulates the host inflammatory response, eventually causing tissue damage.^5^,^6^ Proinflammatory cytokines are considered to be important modulators of inflammation during the initiation and progression of periodontal disease.^7^,^8^ Interleukin (IL)-1, which is a proinflammatory cytokine plays a vital role in immune regulation that causes a variety of inflammatory responses. It has been identified as a periodontal disease marker because of its function as not only an inflammatory mediator but also as a modulator of extracellular matrix and bone.^9^ Although both types of IL-1 (IL-1α and IL-1β) have similar biologic functions, IL-1β is more effective in stimulating bone resorption and occurs more often in periodontitis.^10^

Use of smokeless tobacco products such as *naswar* is widespread in the population of Karachi, contributing to inflammatory, premalignant and malignant diseases of the oral cavity. To the best of our knowledge the effect of *naswar* on periodontal health in terms of salivary IL-1β levels has not been studied so far. Hence, IL-1β levels in association with clinical periodontal parameters will give a better understanding of the effects of *naswar*, specifically on the periodontium.

The objectives of our study were first to assess the clinical periodontal parameters and salivary IL-1β levels in *naswar* users and non-users and then to find a correlation of salivary IL-1β levels with clinical periodontal parameters.

## METHODS

This study was conducted between August 2017 and May 2018. Convenience sampling technique was used for recruitment of participants from the dental outpatient department (OPD) of Ziauddin College of Dentistry, Ziauddin University, Karachi, Pakistan. A WHO Software (“Sample size determination in Health Sciences”) was used for sample size calculation using 0.2% prevalence of *naswar* use as reported by Sajid and Bano (2016) at 95% confidence interval and 80% power of study.^11^ This was a cross-sectional study with eighty-four participants, which were divided into two equal groups. Both genders were included with age’s ≥ 18 years. Individuals who reported consuming *naswar* daily for ≥ 1 year were defined as *naswar* users (Group-1). Controls included those who had never used tobacco in any form (Group-2). Individuals who were present or former users of any other type of tobacco or alcohol products; had systemic inflammatory diseases that could affect the periodontal condition; antibiotics and anti-inflammatory medication usage within 6 months or those who had any periodontal and/or orthodontic procedure in the past 6 months were excluded from the study.

This study was approved by the Ethical Review Committee (ERC) of Ziauddin University, Karachi, Pakistan (Reference code # 0300917ZAOP). It was obligatory for all participants to sign an informed written consent in order to take part in this study.

A questionnaire was used to obtain data from all the participants regarding age, gender, ethnicity, educational status, habits related to *naswar* usage (time period since using, daily frequency, duration of holding a single bolus in the mouth, reason and location of *naswar* placing), oral hygiene practices and whether they considered *naswar* safe for consumption.

For collection of unstimulated whole saliva (UWS) samples, participants were asked to come in early morning hours. They were asked to refrain from eating or drinking at least two hours before saliva collection. For collection of saliva, the patient was first seated on a dental chair and asked to allow UWS to collect in the mouth for five minutes, which was then passively drooled into a graded plastic funnel tube. The samples were stored in a container filled with dry ice and immediately shifted to the Research Laboratory, Ziauddin University. Saliva samples were centrifuged at 2500 rpm (Gemmy Industrial Corp., USA) for 20 minutes at 4°C and the supernatant (around 3ml) was stored at -80°C in multiple aliquots.

A trained examiner (ZA) performed the clinical periodontal examination. The overall *kappa* for intra examiner reliability was 0.84. Full-mouth PI (plaque index), BOP (bleeding on probing), PD (probing depth) and CAL (clinical attachment loss) were measured at six sites per tooth (mesial, middle and distal points on buccal/labial and lingual/palatal surfaces) on all teeth of both arches (excluding third molars).^12^ A University of North Carolina graded probe (Hu Friedy-15) was used to measure PD to the nearest millimeter.

For the quantitative determination of IL-1β concentration in saliva, (Glory Science Co., Ltd) human IL-1β enzyme linked immunosorbent assay (ELISA) kit was used. Manufacturer’s instructions were followed for the indirect ELISA technique being performed. A spectrophotometer (Plate reader stat, Fax-2100, Awareness technology, USA) was used to determine the absorbance by reading the plates at 450nm. This was done twice to check for reproducibility of the results. The detection limit of the kit was 1-20ng/L.

Statistical Package for the Social Sciences (SPSS version 20, Chicago, IL) was used for statistical analysis. After checking normality using Shapiro-Wilk’s test, Mann-Whitney test was run to compare PD, CAL, BI, and PI between *naswar* dippers and controls. Spearman’s correlation was computed to find the correlation between IL-1β with PD, CAL, BI and PI. A p-Value less than .05 was set to show statistical significance of the findings.

## RESULTS

A total of eighty-four male subjects were included in the study (forty-two *naswar* users and forty-two healthy controls). Only two females reported occasional *naswar* usage instead of habitual, hence they were excluded from the study. The mean age of *naswar* users and controls were 33.7 ± 10.8 and 36.4 ± 4.9 years, respectively (Table-I). *Naswar* dippers mostly belonged to the Pathan community (76.2%). Regarding educational status in *naswar* users and non-users, 59.5% and 36.4% were uneducated, respectively. Most common reason for using *naswar* was that they were just addicted to it (52.4%) (Table-I). About 81.1% reported placing it in the premolar-molar vestibular area (right, left or in combination). The mean duration of habit was 12.8 ± 8.1 years, whereas the mean duration of keeping a single *naswar* bolus was 7.2 ± 4.9 minutes (Table-I). An alarming 85.7% of the *naswar* dippers claimed that they considered *naswar* safe for consumption as it had no deleterious effect on the body, particularly oral cavity.

**Table-I:** Characteristics of the study population.

Characteristics	Naswar-dippers (n=42)	Controls (n=42)
Mean age (years)	33.7 ± 10.8	36.4 ± 4.9
***Ethnicity:***		
Pathans	76.2%	61.9%
Sindhi	21.4%	21.4%
Punjabi	2.4%	11.9%
Urdu speaking/Muhajir	0%	4.8%
***Education status:***		
Undergraduate level	39.5%	58.9%
Graduate level	1%	4.7%
Uneducated	59.5%	36.4%
***Reason for using naswar:***		
Just addicted to it	52.3%	NA
Reduce psychological stress	38.1%	
For fresh breathe	4.8%	
For the taste	4.8%
***Position of placement of naswar:***		
Left buccal vestibule	31%	NA
Right buccal vestibule	19%	
Both buccal vestibules	31%	
Adjacent to the lower lip	19%	
Duration of habit (years)	12.8 ± 8.1	NA
Mean daily frequency of *naswar*-dipping	20.3 ± 10.6	NA
Mean duration of keeping a single bolus (minutes)	7.2 ± 4.9	NA
***Oral hygiene maintenance:***		
Brushing (at least once daily)	35.7%	40.4%
Miswak (at least once daily)	40.4%	38.2%
None	23.9%	21.4%

In maintaining oral hygiene, participants reported using brush, miswak and finger (with paste or tooth powder) for cleaning their teeth. Among *naswar* dippers, 35.7% reported brushing and 40.5% miswak use at least once a day (Table-I). It is pertinent to mention that in our part of the world use of Miswak is very popular as a tool for cleaning the teeth. Brushing habit in *naswar* users and controls were 35.7% and 40.4%, respectively. Use of miswak was found to be almost equivalent to brushing habits, that is in *naswar* users and controls it was 40.4% and 38.2%, respectively. About 23.9% of *naswar* dippers and 21.4% of controls reported that they did not use any technique for maintaining oral hygiene (Table-I).

CAL, PD of 4mm and in 5-6mm range was significantly higher in *naswar* dippers than controls. Difference in clinical parameters among the two groups was determined by applying Mann-Whitney U test. There was no significant difference in PI, BI, PD > 7mm and the number of missing teeth (Table-II).

**Table-II:** Mean and range of clinical periodontal parameters among naswar dippers and control group.

	Naswar-dippers	Controls
Salivary IL-1β (ng/L)	8.3 ± 3.1*	2.3 ± 1.1
PD 4mm (%)	9.3 (0.0-42.9)*	4.4 (0.0-32.6)
PD 5-6mm (%)	5.0 (0.0-33.9)*	2.3 (0.0-28.3)
PD >=7mm (%)	0.1 (0.0-1.6)	0.3 (0.0-8.0)
CAL (mm)	2.9 (1.3-5.7)**	2.1 (1.0-7.6)
Plaque Index (%)	3.1 (0.0-13.4)	2.6 (0.0-9.1)
Bleeding Index (%)	2.7 (0.0-11.6)	2.4 (0.0-11.1)
Missing teeth	11.1 (0.0-48.0)	5.5 (0.0-42.0)

* P-value < 0.05,

** P-value < 0.001

Mann-Whitney U test applied

Mean Interleukin 1-β levels in *naswar* dippers and controls was 8.3 ± 3.1 ng/L and 2.3 ± 1.1 ng/L, respectively (Table-II). There was a significant positive correlation between values of CAL (P<0.01) PD 4mm and 5-6 mm (P<0.05) with salivary Interleukin 1-β levels after applying Spearman’s correlation test (Table-III).

**Table-III:** Correlation of Interleukin 1-β levels with clinical periodontal parameters.

	IL-1β level	

	Correlation value	P – value
PD 4mm (%)	0.248*	0.023
PD 5-6mm (%)	0.254*	0.020
PD >=7mm (%)	0.131	0.236
Clinical attachment loss (mm)	0.332**	0.002
Plaque Index (%)	-0.072	0.515
Bleeding Index (%)	-0.027	0.805

* P-value < 0.05,

** P-value < 0.005

Spearman’s correlation test applied

## DISCUSSION

To the best of our knowledge after going through published literature, this is the first study assessing salivary IL-1β level in *naswar* users. Multiple studies have shown that use of STPs including gutka, snus, shammah etc, have harmful effects on the oral cavity eventually leading to malignant lesions.^13^, ^14^ Studies show that STPs use among Pakistani population is more in males then in females, and there is even a larger difference among the rural population.^15^,^16^ As mentioned before the use of *naswar* has become widespread and is gaining popularity, therefore researchers are now focusing on studies reporting the effects of *naswar* on the oral cavity. A study assessing cytomorphometric changes in buccal smear of *naswar* users, reported dysplastic changes in the oral mucosa, hence pointing to the potential of causing oral premalignant/malignant lesions as well.^17^ Recent studies have also associated *naswar* usage with alveolar bone loss and concurrent aggravation of clinical periodontal parameters.^13^, ^18^ Contrary to our findings, a study assessed IL-1β levels in serum of *naswar* dippers which showed the levels to be significantly lowered in *naswar* users as compared to healthy controls.^11^

Present study showed that IL-1β level were raised in the saliva of *naswar* users as compared to the healthy controls.IL-1β levels in both GCF and saliva of other type of STP users, was also found to be raised; emphasizing the point that IL-1β plays a critical role in periodontitis and that its levels were increased more when associated with a risk factor such as use of STPs.^14^,^19^,^20^ This could be due to the fact that *naswar* users have a poorer periodontal condition as shown by elevated PD and CAL. An interesting finding in our study was that though there was very significant difference in CAL and PD of *naswar* users as compared to non-users, the alteration in PI, BOP and missing number of teeth wasn’t statistically significant. This could be attributed to the fact that most of the study population belonged to low socioeconomic status and were mostly uneducated; hence there was poor oral hygiene maintenance in the healthy controls as well. Similarly a justification for an increased number of missing teeth could be due to these patients opting for extraction rather than costly but appropriate treatments such as fillings and root canals.

GCF is the primary source of periodontitis-associated cytokines and host-derived enzymes; however these substances will ultimately enter into the saliva.^21^,^22^ Though, mostly GCF is taken as a sample medium, it is very difficult because:

1. It is a technique sensitive method, as collection requires skilled person who can prevent contamination of the GCF strips or paper points from saliva;
2. There are chances of bleeding when collecting GCF which again contaminates the sample;
3. GCF strips or paper points require an added electronic Periotron device to determine the volume obtained, which is not available easily;
4. Collecting GCF through micropipette may cause trauma to the gingiva as well. In addition to these, saliva collection is less troublesome and several samples can be collected without causing any distress to the patient, hence it was the selected medium for this study.^21^

In this study we attempted to see the use of *naswar* among various ethnicities residing in Karachi city namely Urdu speaking/Muhajir, Punjabi, Sindhi and Pathans as previously *naswar* dipping was considered to be specific to those belonging from Pathan ethnic group. Though they are still the most common users of the product, a rise in usage is seen in other communities as well which is also shown in other studies as well.^15^

It is important for health care providers to have adequate knowledge regarding the detrimental effects of *naswar* usage so they can convey that knowledge to those who use this STP. There’s an alarming myth among the *naswar* dippers that it is safe for consumption as compared to other STPs and has no detrimental effects on the oral cavity or to the body internally. Only 14.3% of the *naswar* users in our study believed that it may have detrimental effects on the oral tissues and may cause oral cancer. Due to increase in evidence of potential harmful effects of *naswar*, there is a need for the health department to take action against use of *naswar*, as they have recently imposed sanctions on sale of gutka and betel nuts in the country.

### Limitation of the study

Answers to the questions regarding demographics and systemic illnesses were based on self-reporting and no test/examination was done to actually determine if the participants were systemically healthy or not. This is a limitation of the study, as systemic diseases can have adverse effects on the periodontium and alter IL-1β levels as well.

## CONCLUSION

Within the perimeters of this study, it can be concluded that IL-1β levels in saliva are raised and clinical periodontal conditions are worse in habitual *naswar* users as compared to non-users.

### Future Recommendation

Levels of IL-1β might be checked both in saliva and GCF of *naswar* users, thus drawing a comparison between the effectiveness of the two mediums.
